# Accuracy of autofluorescence in diagnosing oral squamous cell carcinoma and oral potentially malignant disorders: a comparative study with aero-digestive lesions

**DOI:** 10.1038/srep29943

**Published:** 2016-07-15

**Authors:** Xiaobo Luo, Hao Xu, Mingjing He, Qi Han, Hui Wang, Chongkui Sun, Jing Li, Lu Jiang, Yu Zhou, Hongxia Dan, Xiaodong Feng, Xin Zeng, Qianming Chen

**Affiliations:** 1 State Key Laboratory of Oral Diseases, West China Hospital of Stomatology, Sichuan University, Chengdu, Sichuan 610041, China; 2 Department of Oral Medicine, School of Stomatology, Capital Medical University, Beijing 100050, China

## Abstract

Presently, various studies had investigated the accuracy of autofluorescence in diagnosing oral squamous cell carcinoma (OSCC) and oral potentially malignant disorders (OPMD) with diverse conclusions. This study aimed to assess its accuracy for OSCC and OPMD and to investigate its applicability in general dental practice. After a comprehensive literature search, a meta-analysis was conducted to calculate the pooled diagnostic indexes of autofluorescence for premalignant lesions (PML) and malignant lesions (ML) of the oral cavity, lung, esophagus, stomach and colorectum and to compute indexes regarding the detection of OSCC aided by algorithms. Besides, a *u* test was performed. Twenty-four studies detecting OSCC and OPMD in 2761 lesions were included. This demonstrated that the overall accuracy of autofluorescence for OSCC and OPMD was superior to PML and ML of the lung, esophagus and stomach, slightly inferior to the colorectum. Additionally, the sensitivity and specificity for OSCC and OPMD were 0.89 and 0.8, respectively. Furthermore, the specificity could be remarkably improved by additional algorithms. With relatively high accuracy, autofluorescence could be potentially applied as an adjunct for early diagnosis of OSCC and OPMD. Moreover, approaches such as algorithms could enhance its specificity to ensure its efficacy in primary care.

The global incidence rate of oral cancer is 8.2 per 100,000 annually for males and 2.8 per 100,000 annually for females^1^. More than 90% of oral cancers are oral squamous cell carcinomas (OSCCs), which are one of the most common malignant tumors and are more prevalent in South-Central Asia and in Central and Eastern Europe^1^,^2^. Owing to the unapparent symptoms in early stages and delayed diagnosis^3^,^4^, OSCC tends to be detected at advanced stages with high mortality, accounting for approximately 300,000 cases of OSCC and 145,000 deaths in 2012^12^. Despite treatment advancements, the 5-year survival rate for OSCC patients has remained poor over the past three decades^5^. However, if OSCC is diagnosed early (stage I-II) and effective treatment is administered, a 5-year survival rate of approximately 80% is obtainable compared with only 20% for those detected at advanced stages (stage III-IV)^5^. Therefore, early detection is crucial to help improve the survival rate of OSCC^6^.

Usually, patients initially present to general dental practice when they have oral discomfort. Thus, as frontline health workers, general dentists bear the responsibility for early screening of oral abnormalities^6^. Furthermore, dental practitioners play a vital role in making correct decisions about the lesions, by which unnecessary or delayed referrals could be avoided and the mortality of OSCC could be considerably reduced^5^,^7^,^8^. In most cases, OSCC is preceded by oral potentially malignant disorders (OPMD) such as oral leukoplakia and oral submucous fibrosis^9^,^10^, so the early detection and management of epithelial dysplasia in OPMD is an important preventative step against malignant transformation^11^; Moreover, since dysplastic and neoplastic lesions of the oral cavity are readily accessible and the mucosal changes are frequently visible, OSCC and dysplasia are both promising candidates for routine screening^5^. According to one large population-based study in India, periodic visual screening of the oral cavity has contributed to a reduction of 32% in mortality during a period of 9 years. Therefore, dentists are encouraged to commit to OSCC screening as a routine daily practice^12^.

The current guideline for detecting OSCC and OPMD recommends a conventional oral examination (COE), which involves visual examination and tactile palpation under white light^11^. A Cochrane review stated that insufficient evidence was suggested for the application of COE in OSCC screening programs within a low-risk population^13^; a systemic review also illustrated the ineffectiveness of COE in detecting dysplasia and OSCC^14^. One main limitation is the possible inability of general dentists to differentiate between benign and high-risk lesions, as early stage of advanced lesions may not present with typical features^7^. Apart from COE, tissue biopsy is only suggested for clinically suspicious lesions^15^. Although histological biopsy is recognized as the gold standard for the diagnosis of OSCC and dysplasia in OPMD, it is invasive, time-consuming and painful^16^.

Given the concerns about COE and biopsy, alternative methods should be developed for the early identification of OSCC and OPMD, particularly for the primary care^5^,^17^. As is reported, the wide application of exfoliative cytology in the early diagnosis of cervical cancer has decreased its mortality remarkably^18^. Hopefully, analogous early diagnostic tools with high accuracy could be applied to relieve the global disease burden of OSCC. Currently, various non-invasive diagnostic methods have emerged^8^. As a novel approach for early cancer diagnosis and a representative light-based detection system, autofluorescence is a non-invasive, convenient and real-time device^16^,^19^.

In an attempt to apply autofluorescence in the inspection of premalignant lesions (PML) and malignant lesions (ML), understanding the biological basis of autofluorescence is indisputably essential. In brief, the diagnostic potential of autofluorescence lies in the ability to probe alterations in tissue structure and metabolism that occur during malignant progression^10^. When normal tissue is illuminated by the excitation light of certain wavelength, some molecules in the tissue, called fluorophores, would absorb photons and emit lower energy photons that could be detected as fluorescence from the mucosal surface^20^. And the dominant fluorophores, responsible for autofluorescence signals, include reduced nicotinamide adenine dinucleotide (NADH) and flavin adenine dinucleotide (FAD) in the epithelium together with collagen matrix and elastin in the stroma^20^. However, in PML and ML, loss or weakening of autofluorescence would appear, mainly attributed to the breakdown of collagen matrix and elastin and the altered metabolism (decrease of NADH and FAD) during neoplastic progression^10^. Further, other metabolic or structural alterations in PML and ML are also associated with the loss or alteration of autofluorescence. Specifically, accumulated porphyrins might lead to an increased red fluorescence in addition to changes in the green one^20^; besides, massive heme formed by the porphyrins, and the structural changes (epithelial thickening, increased nuclear size, hyperchromatin and increased microvascularity) would contribute to the increased absorption and/or scattering of light, eventually reducing the detectable autofluorescence^10^,^19^,^21^.

Over the past three decades, the accuracy of autofluorescence has been widely evaluated in the diagnosis of OSCC, lung cancer (bronchiogenic carcinoma), esophageal cancer, stomach cancer, colorectal cancer and PML^16^,^22^,^23^,^24^,^25^, with PML representing dysplastic or neoplastic lesions^21^,^26^,^27^. After confirmation of its reliability, some autofluorescence technology such as LIFE has been used for routinely endoscopy^28^. However, inconsistent conclusions have been reached in various studies exploiting different autofluorescence technology.

In previous studies, Lane *et al*. had achieved relatively high diagnostic accuracy of autofluorescence when used a simple device for visualisation of oral-cavity fluorescence^21^, while Farah *et al*. obtained an opposite result aided by a representative of autofluorescence termed the Visually Enhanced Lesion Scope (VELScope; LED Dental, Vancouver, BC, Canada)^29^. Interestingly, Roblyer *et al*. developed an algorithm with autofluorescence to detect OSCC and OPMD with ideal sensitivity and specificity, implying the potential role of an additional algorithm for improving the accuracy of autofluorescence^20^. One systemic review on the use of autofluorescence to diagnose OPMD and OSCC has implied that it was more suitable for specialist clinics than for primary care^30^. In addition, a recent Cochrane review appraised the diagnostic accuracy of light-based detection for OPMD and OSCC, and its sensitivity and specificity were estimated as 0.91 and 0.58, respectively. They reported that there was a lack of evidence to support the replacement of histological biopsy by light-based detection combined with COE^31^. However, only 5 of those studies were about autofluorescence and their inclusion criteria was different from our study.

Thus far, although community dentists are in greater need of a screening tool for OSCC and OPMD, a limited number of studies have been performed to evaluate autofluorescence for routine screening based on the general population. Huff *et al*.^32^, as the first to report the efficacy of autofluorescence for screening in a community practice setting, showed that there was higher yield of mucosal abnormalities for VELscope than COE, and 83% of these abnormalities found by VELscope were epithelial dysplasia. Conversely, McNamara *et al*.^33^ concluded that COE was more valid than the VELscope findings since the FP of VELscope remain a concern in general practice. Nevertheless, several studies have demonstrated the effectiveness of autofluorescence in discerning OSCC or dysplastic lesions from clinically innocuous lesions that were missed by COE^29^,^34^. Moreover, aiming at overcoming the low specificity of autofluorescence, two studies have integrated autofluorescence into decision-making protocols for general dentists with encouraging outcome^17^,^35^.

As distinct conclusions are made by numerous studies about the accuracy of autofluorescence for OSCC and OPMD and its applicability in general practice, a more thorough study is needed. In this study, based on a meta-analysis and the *u* test method, by making a comparison with other common aero-digestive PMLs and MLs and calculating the pooled diagnostic indexes of autofluorescence for the 5 aforementioned parts, we attempted to evaluate the overall diagnostic accuracy of autofluorescence for OSCC and OPMD and discuss its applicability as an adjunct in general dental practice. Further, a contrast of using autofluorescence alone or with proper algorithms for OSCC and OPMD was performed to appraise the impact of additional algorithms. To the best of our knowledge, no study on this issue has been previously conducted.

## Materials and Methods

### Search strategy and study selection

Two reviewers (X. Luo and H. Xu) independently conducted a literature search using the electronic databases PubMed, Ovid Medline and Embase (updated to November 18, 2015). There was no language restriction, and we mainly focused on English literature. The search terms were listed below: “Autofluorescence” or “spectrometry, fluorecence” or “VELscope”and “Leucoplakia, Oral” or ”precancerous conditions” or “Mouth Neoplasms”, “Autofluorescence” or “spectrometry, fluorecence” and “precancerous conditions” or ”lung neoplasms” or ”stomach neoplasms” or ”esophageal neoplasms” or ”colorectal neoplasms”. The reference lists of all of the included studies were also searched for possible inclusion. The inclusion criteria used in the selection of literature for our study were as follows: (1) Adopting VELscope or other autofluorescence tools alone or with algorithms as diagnostic tools of OPMD and OSCC, and utilising autofluorescence tools alone or with algorithms for the diagnosis of PML and ML of the lung, esophagus, stomach, or colorectum; (2) lesions of the study population were clinically diagnosed as PML or suspicious ML of the oral cavity, lung, esophagus, stomach, or colorectum; (3) histological results are the gold standard for diagnosis, and positive histological findings should include mild to moderate to severe epithelial dysplasia or mucosa low-grade to high-grade neoplasia, carcinoma *in situ*, or a malignant tumor. Otherwise, these results should be defined as negative histological results; (4) only original clinical trials of human would be included; (5) providing sufficient data to construct a 2 × 2 table to calculate the sensitivity and specificity.

### Data extraction and quality assessment

Data extraction was performed by the same reviewers ((X. Luo and H. Xu)) independently. Any discrepancies were resolved by discussion with a third author (Q. Chen). The following data were collected from each study: First author’s name, publication year, country, tumor type, sample size, true positive (TP), false positive (FP), false negative (FN), true negative (TN), sensitivity, and specificity. Initially, the standards for reporting diagnostic accuracy (STARD) is utilised to evaluate the methodological quality of the included studies^36^,^37^. Moreover, the quality assessment for studies of diagnostic accuracy (QUADAS-2) tool, as another powerful quality-assessing tool, was applied to evaluate the risk of bias and applicability concerns of these included studies in 4 key domains of patient selection, index test, reference standard, and flow and timing^38^,^39^.

### Statistical analyses

The standard statistical methods, recommended for meta-analysis of diagnostic test evaluations, were used for assessing the accuracy of autofluorescence for detecting PML and ML of the oral cavity, lung, esophagus, stomach and colorectum and evaluating the accuracy of using autofluorescence alone or with algorithms for OSCC and OPMD. Upon TP, FP, FN, TN results derived from each original study, the following indexes regarding the diagnostic accuracy of autofluorescence for PML and ML of a certain area of the body were calculated for the according study: sensitivity (Sen), specificity (Spe), positive likelihood ratio (PLR), negative likelihood ratio (NLR) and diagnostic odds ratio (DOR)^40^. Then, aimed to calculate the pooled estimates of sensitivity and specificity, together with their 95% confidence intervals (CI), and present sensitivity and specificity more intuitively, the forest plots were plotted, each segment representing the effect size and confidence intervals of every study in the coordinate system^40^. Similarly, the pooled PLR, NLR and DOR were computed by depicting their forest plots. The threshold effect, usually arising when different cut-off points are applied to define a positive result of a diagnostic test among the included studies, was detected by calculating the Spearman’s correlation coefficient based on the aforementioned sensitivity and specificity, with p < 0.05 indicating existence of threshold effect^40^. If threshold effect is implied, Hierarchical summary receiver operating characteristic (HSROC) curves, based on sensitivity and specificity of autofluorescence for every site, could be depicted, with a hope to characterize the overall diagnostic performance of autofluorescence for each anatomical site by computing the area under the curve (AUC)^41^. Moreover, the wind rose, a frequently used chart in meteorology, was taken as a reference to reflect the overall diagnostic accuracy of autofluorescence among PML and ML of the oral cavity and the other 4 parts more intuitively, containing the 6 indexes (pooled Sen, Spe, PLR, NLR, DOR and AUC) calculated above. Besides, the maximum values of the 6 indexes were all standardized as 1 to allow a plain understanding of these results. Specifically, in terms of each diagnostic index except for NLR, farther distance from the center of the wind rose means higher accuracy, while it is just the opposite for NLR. To contrast a certain index of autofluorescence among PML and ML of the 5 body parts and to compare the diagnostic index of applying autofluorescence alone or with algorithms, a *u* test was administered^42^. Then, Bonferroni was adopted as a correction to reduce I type error, and the inspection level was 0.0125 and 0.05, respectively^43^.

The chi-squared-based *Q* test and the inconsistency index *I*^*2*^ were used to calculate the inter-study heterogeneity^44^,^45^. Heterogeneity among studies would be suggested when the *Q* test was significant (*p* < 0.05) or *I*^*2*^ > 50%; thereafter, the random-effect model (DerSimonian–Laird method) would be chosen to conduct the meta-analysis. Otherwise, the fixed-effect model would be selected^44^,^45^. Meta-regression was then administered to investigate the source of observed heterogeneity^40^.

Besides, Begg’s funnel plot and Egger’s test were used to test publication bias and each circle in the funnel plot means one study, with symmetrical inverted funnel in the chart representing none publication bias and asymmetrical one signifying publication bias^46^,^47^.

All of the analyses were performed using the following statistical software programs: STATA 11.0 (Statacorp, Texas, USA) and Revman 5.0 (The Cochrane Collaboration). All tests were two-sided, and *p* < 0.05 was considered statistically significant.

## Results

### Quality of reporting and study characteristics

**Figure 1:**
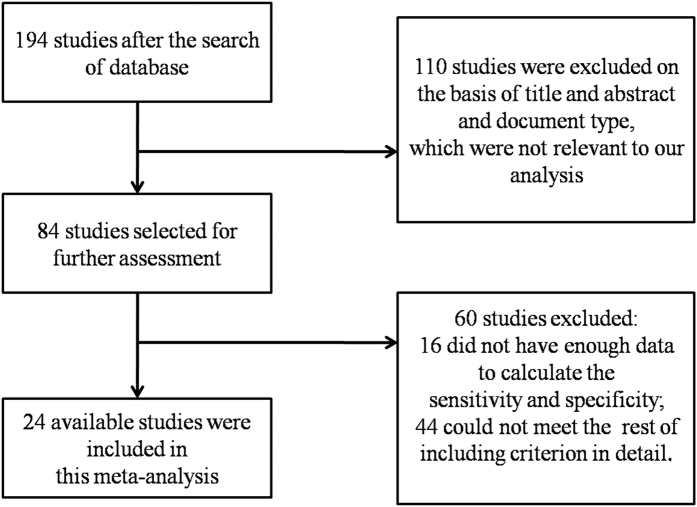
Flow diagram shows the selection process of eligible articles that applying autofluorescence to diagnose OSCC and OPMD. OSCC: oral squamous cell carcinoma; OPMD: oral potentially malignant disorders.

We found 194, 197, 61, 46, and 117 articles that applied autofluorescence to diagnose OSCC, lung cancer, esophageal cancer, stomach cancer and colorectal cancer, respectively, with an available PML. After identifying the titles and abstracts, a full-text assessment, and strict filtering according to the inclusion criteria, the corresponding numbers of included studies were 24,^16^,^19^,^20^,^21^,^29^,^48^,^49^,^50^,^51^,^52^,^53^,^54^,^55^,^56^,^57^,^58^,^59^,^60^,^61^,^62^,^63^,^64^,^65^,^66^ 25,^22^,^67^,^68^,^69^,^70^,^71^,^72^,^73^,^74^,^75^,^76^,^77^,^78^,^79^,^80^,^81^,^82^,^83^,^84^,^85^,^86^,^87^,^88^,^89^,^90^ 12,^23^,^91^,^92^,^93^,^94^,^95^,^96^,^97^,^98^,^99^,^100^,^101^ 9,^24^,^102^,^103^,^104^,^105^,^106^,^107^,^108^,^109^ and 19,^25^,^110^,^111^,^112^,^113^,^114^,^115^,^116^,^117^,^118^,^119^,^120^,^121^,^122^,^123^,^124^,^125^,^126^,^127^, respectively. The search and selection process were described as a flow diagram in Fig. 1 and Supplementary Fig. S1. A summary of the main characteristics of the included diagnostic studies was presented in Table 1 and Supplementary Table S1. All of the research that used autofluorescence for OSCC and OPMD were prospective studies, while for studies detecting the PML and ML of the lung, esophagus, stomach and colorectum, only 1, 1, 1, and 1 study was found to be a retrospective study^90^,^99^,^105^,^125^. Most of the studies treated autofluorescence loss or color change as a positive result, whereas 8, 5, 4, and 9 studies on the detection of PML and ML of the oral cavity, esophagus, stomach and colorectum, respectively, applied algorithms to discriminate positive or negative results.

As for the reporting quality of the diagnostic studies, the STARD scores of the corresponding 24, 25, 12, 9, and 19 studies were all ≥17 and were of relatively high quality. Aimed to further assess the quality of the diagnostic trials, the QUADAS-2 tool was used. Based on these studies, each of the 7 components was graded as “low risk of bias” (LR), “unclear risk of bias” (UR), “high risk of bias” (HR) and “low concern” (LC), “high concern” (HC), and “unclear concern” (UC). Among the 24 studies for OSCC and OPMD, HR results of patient selection were yielded in 8 studies, 6 of the 8 reports were associated with high-risk patients who were histologically diagnosed as OSCC^16^,^21^,^50^,^53^,^57^,^60^. In terms of the remaining 6 components for OSCC and OPMD, LR, UR, LC and UC were generally displayed. Overall, the qualities of these studies were shown in Table 2 and Supplementary Table S2.

### Diagnostic accuracy of autofluorescence for OSCC and OPMD

**Figure 2:**
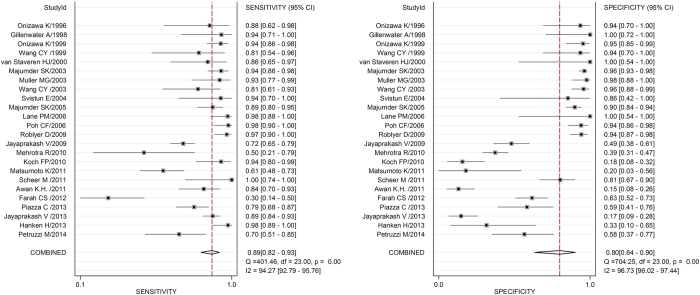
Forest plots of sensitivity and specificity of studies that employing autofluorescence in the diagnosis of OSCC and OPMD. The solid circles indicate estimates of sensitivity and specificity for each study, and the size of each solid circle represents the sample size of each study. The error bars are 95% confidence intervals. OSCC: oral squamous cell carcinoma; OPMD: oral potentially malignant disorders.

To assess the diagnostic accuracy of autofluorescence for OSCC, lung cancer, esophageal cancer, stomach cancer and colorectal cancer and their PML, Fig. 2 and Supplementary Fig. S2 showed the forest plots of the pooled sensitivity and specificity of the corresponding 24, 25, 12, 9, and 19 studies; moreover, the forest plots of sensitivity and specificity on the use of autofluorescence alone or with algorithms for OSCC and OPMD were also depicted in Supplementary Fig. S3 and S4, respectively. Analogously, the pooled PLR, NLR and DOR were achieved by the same methods.

### Comprehensive evaluation of diagnostic performance of autofluorescence for OSCC and OPMD

**Figure 3:**
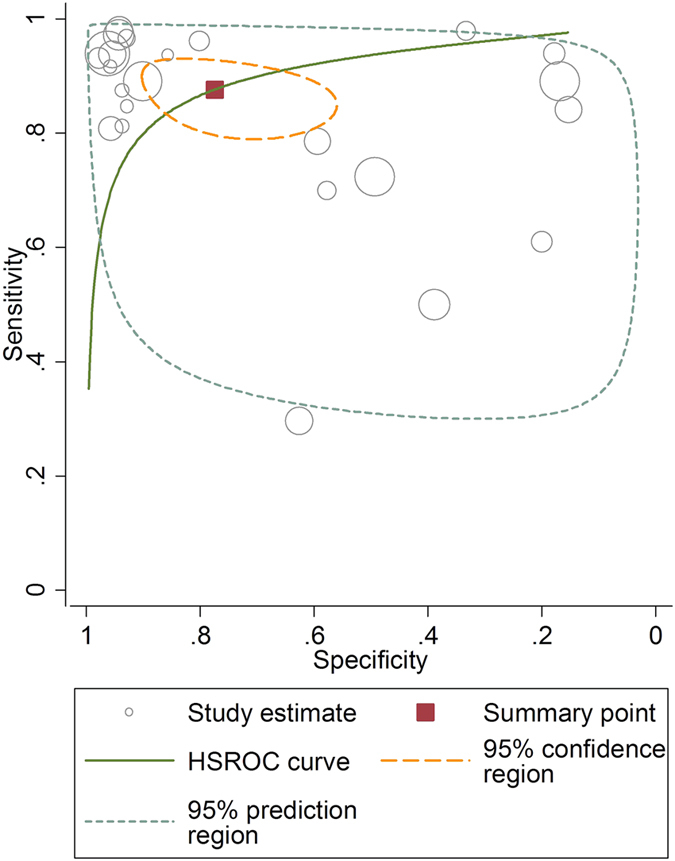
Hierarchical summary receiver operating characteristic curve (HSROC) of studies utilising autofluorescence for the detection of OSCC and OPMD. Each empty circle represents one study, and the size of every circle indicates the sample size of each study. OSCC: oral squamous cell carcinoma; OPMD: oral potentially malignant disorders.

After computation of Spearman’s correlation coefficient upon the sensitivity and specificity of according studies, existence of threshold effect (all *p* < 0.05) was generally revealed in studies with regard to diagnosis of the 5 various body sites. Thus, the HSROC curves were depicted in Fig. 3 and Supplementary Fig. S5. As a descriptive index of the HSROC curve, AUC, pertaining to the overall diagnostic accuracy of autofluorescence for the various area of body, were computed based on the curves.

Afterwards, pooled estimates of the 5 indexes (Sen, Spe, PLR, NLR and DOR) and AUC of these studies regarding utilising autofluorescence in the detection of PML and ML of 5 body sites were shown in Table 3. Furthermore, the 6 indexes on the application of autofluorescence alone or with algorithms for OSCC and OPMD were also separately displayed in Table 4.

To seek a comprehensive evaluation of these 6 indexes regarding the diagnostic value of autofluorescence for PML and ML of the 5 anatomical areas from another perspective, a wind rose picture derived from meteorology was depicted (Supplementary Fig. S6). It indicated that the AUC of PML and ML of the 5 areas were all relatively high, among which, the overall diagnostic accuracy of autofluorescence for OSCC and OPMD was slightly inferior to colorectal cancer along with its PML, but was superior to the PML and ML of the other 3 areas, whereas the values of the other 5 indexes varied in terms of PML and ML of diverse sites. Particularly, optimal specificity and PLR were suggested when autofluorescence was applied to detect OSCC and OPMD.

### *U* test of 6 diagnostic indexes

A *u* test of the 6 indexes (pooled Sen, Spe, PLR, NLR, DOR and AUC) that implied the diagnostic accuracy of autofluorescence among PML and ML of the 5 areas was conducted, indicating that statistical significance only lies in the comparison of the AUC between detecting PML and ML of the oral cavity and esophagus. Subsequently, another *u* test result concluded that a significant difference could be observed in Spe, PLR, DOR and AUC (Table 4), suggesting that better accuracy for detecting OSCC and OPMD could be achieved with the use of algorithms rather than autofluorescence alone.

### Heterogeneity test

Autofluorescence was used to diagnose PML and ML of 5 anatomic sites. Thus, we divided this study into 5 sections. The *I*^*2*^ of the 6 indexes including Sen, Spe, PLR, NLR, DOR and AUC of all 5 sections was greater than 50%, and all *Q* test results were *p* < 0.05, which implied a significant heterogeneity across the included studies for all 5 sections. The random effects model was chosen to calculate the 6 pooled indexes. A meta-regression analysis was performed to identify the possible cause of heterogeneity according to the following study characteristics: population characteristics, study design, sample size, threshold effect and lack of blinding. However, none of the above analysed factors were significantly different (all *p* > 0.05).

### Evaluation of publication bias

**Figure 4:**
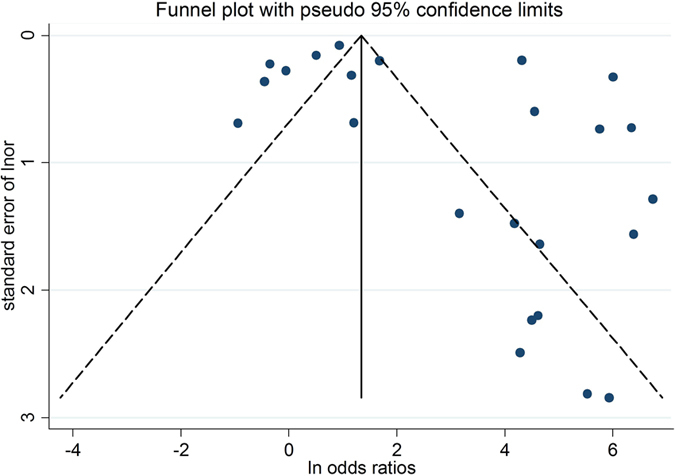
Begg’s funnel plot for the evaluation of potential publication bias of studies that applying autofluorescence to diagnose OSCC and OPMD. Each solid circle indicates one study; OSCC: oral squamous cell carcinoma; OPMD: oral potentially malignant disorders.

The Begg’s funnel plot and Egger’s test were selected to explore publication bias of the 5 sections, and all results of the Egger’s test were *p* > 0.05, each funnel plot manifesting as symmetrical inverted funnel-shaped figure. Both analyses indicated insignificant publication bias (Fig. 4 and Supplementary Fig. S7).

## Discussion

Presently, early diagnosis is supposed to improve the outcome of OSCC by general dentists. While COE, as an available method, seems to be helpless particularly for inexperienced dental practitioners^128^. Thereby, adjunct diagnostic aids are desperately needed by primary care workers to facilitate the early detection of OSCC and dysplasia. Over the past three decades, the diagnostic performance of autofluorescence has been explored in OSCC and OPMD in several studies with conflicting results, also in 4 common aero-digestive lesions^59^,^60^,^61^. Therefore, our study was conducted to further investigate its overall diagnostic accuracy and to discuss its application stability in 5 anatomic parts in the hope of recommending it as a diagnostic aid in general dental practice. After calculating the pooled diagnostic indexes of autofluorescence for OSCC and OPMD along with the PML and ML of the lung, esophagus, stomach and colorectum, an overall good accuracy of autofluorescence for detecting OSCC and OPMD was indicated, with a pooled sensitivity and specificity of 0.89 and 0.80, respectively. In addition, the relatively preferable accuracy of autofluorescence for all of the 5 anatomical parts has implied its application stability and potential role in primary care. As our included studies were generally based on non-primary care settings, autofluorescence is presently more proper for specialists. However, based on our included studies, proper algorithms could be combined with autofluorescence to enhance its specificity for its promising use in primary care.

Aiming at initially evaluating the accuracy of autofluorescence for OSCC and OPMD, we calculated its pooled Sen, Spe, PLR, NLR and DOR. According to the forest plots, the pooled sensitivity of autofluorescence for detecting OSCC and OPMD is as high as 0.89, which is better than PML and ML of the esophagus and stomach, slightly worse than that of the colorectum and equal to that of lung, although no statistically significant difference was found. In addition, the pooled specificity of autofluorescence for detecting OSCC and OPMD was 0.8, although not high enough to reach statistical significance, it is optimal among the 5 areas. Besides, when the pooled sensitivity and specificity of using autofluorescence alone or with algorithms were separately computed, an encouraging result was indicated, presented as 0.88 and 0.62, 0.92 and 0.95, respectively.

PLR and NLR are more clinically significant and practical for the measurement of diagnostic accuracy. In this study, the PLR and NLR values for detecting OSCC and OPMD were 4.54 and 0.14, respectively, suggesting that patients with OSCC or dysplastic lesions have about a 4.54-fold higher chance of being autofluorescence-positive compared to those without them, while the chance of having OSCC or dysplastic lesions in autofluorescence-negative patients is theoretically 14%. The PLR of the autofluorescence for detecting OSCC and OPMD is optimal, although it is not statistically significant. The NLR of autofluorescence for detecting OSCC and OPMD is better than PML and ML of the lung, esophagus and stomach, and slightly inferior to the colorectum, with no statistical significance indicated. DOR has combined sensitivity and specificity into a single number, with higher values represent better diagnostic performance^129^. In our study, the DOR for detecting OSCC and OPMD is 32.37 with good accuracy, slightly lower than PML and ML of the colorectum and greater than the other 3 areas. Additionally, a significant increase can be observed in the PLR, DOR of autofluorescence for identifying OSCC and OPMD assisted by proper algorithms, displayed as 17.28 and 194.11.

According to the AUC values of the HSROC curves, a better overall diagnostic accuracy of autofluorescence for PML and ML of the oral cavity and colorectum was revealed compared to the lung, esophagus and stomach, and a statistically significant difference was only indicated between the oral cavity and esophagus. Although the diagnostic accuracy of autofluorescence for OSCC and OPMD was slightly less than colorectal cancer and its PML, the AUC of the former was also greater than 0.9, which represents relatively good overall diagnostic accuracy. Further, the AUC of applying autofluorescence alone or with algorithms were individually estimated as 0.85 and 0.98 with statistical significance.

Aiming at understanding our results more vividly, the wind rose picture was delineated, revealing that the overall diagnostic accuracy of autofluorescence for OSCC and OPMD is superior to PML and ML of the lung, esophagus and stomach and is only inferior to the colorectum. In addition, the sensitivity and specificity for the 5 body parts fluctuated between 0.78 to 0.91 and 0.63–0.80, respectively. The stable and relatively high accuracy of autofluorescence across the five parts has confirmed its efficacy under diverse conditions, suggesting its potential role in primary care.

Across our included literatures, the sensitivity of autofluorescence for detecting OSCC and OPMD ranged from 30% to 100%; high sensitivity was indicated in the majority of studies except for low values in 3 studies^29^,^60^,^62^. Conversely, low specificity varying between 15.3% to 63% was implied in 10 of our included studies from 2009 to 2014^19^,^29^,^58^,^59^,^60^,^62^,^63^,^64^,^65^,^66^. Other studies also reported low specificity by emphasizing its inability to discriminate benign lesions from malignant or premalignant mucosal conditions as well as a high rate of false positive results^11^,^63^,^130^. Meanwhile, Balevi *et al*. stated that adoption of autofluorescence as a cancer-screening device for general dentists was presently premature^131^. In addition, as all of our included studies regarding identifying OSCC and OPMD are based on non-primary care settings and 6^16^,^21^,^50^,^53^,^57^,^60^ of these studies are on high-risk patients who were previously diagnosed as OSCC in the cancer clinics, the extrapolation of autofluorescence alone to the care remains a significant concern, and it is thought to be more valuable in the hands of specialists by most studies^30^,^31^,^131^. In specialist clinics, Poh *et al*. used it in the identification of tumor margins during the operation^16^. Kois *et al*. suggested that autofluorescence could assist in deciding the best biopsy area^130^. However, such a tool caters more to dental practitioners to uncover early OSCC and dysplasia in clinically innocuous lesions^6^.

Despite the fact that it seems unlikely to be widely applied in general dental practice mainly due to low specificity, fortunately, some available approaches could be combined with autofluorescence to facilitate its application within the primary care environment. Of the 24 studies regarding the use of autofluorescence for diagnosing OSCC and OPMD, 8 studies incorporated proper algorithms into the analysis of autofluorescence^20^,^49^,^50^,^52^,^53^,^54^,^55^,^57^, with a pooled sensitivity and specificity estimated to be 0.92 and 0.95, respectively. However, after separately calculating the diagnostic indexes of the remaining 16 studies, sensitivity and specificity were found to be 0.88 and 0.62, respectively. Thereby, the method of combining algorithms with autofluorescence could be generalized to low-risk groups to improve accuracy, and more similar studies in general dental settings are needed to warrant its efficacy.

Although inconsistent sensitivity and specificity of autofluorescence for OSCC and OPMD were presented in our included studies, primarily depending on both the different excitation and emission properties of the fluorophores and the diverse ability to discriminate PML and ML from normal regions based on the utilisation of various wavelength of excitation/emission light and algorithms, optimal accuracy were obtained in some of our included studies, either using autofluorescence alone or with proper algorithm. Thus, it indicates the potential of seeking a more suitable wavelength of excitation/emission light and algorithm, even individually designed for benign, dysplastic and malignant oral lesions, on the basis of these previous studies, to significantly enhance the accuracy of autofluorescence for detecting OSCC and OPMD. For instance, Pavlova I *et al*. proposed that in their study, excitation wavelengths in the UV range may improve the accurate diagnosis of PML and ML of oral cavity^132^.

Additionally, as the first to introduce autofluorescence into community dental practices, Laronde *et al*. devised a stepwise protocol, including patient history, visual screening examination, lesion risk assessment and direct fluorescence, to guide 18 general dentists to apply autofluorescence in their offices. Besides, a return for a 3-week reassessment was emphasized to reduce FP results on initial visits. Finally, it highlighted that combination of this protocol and autofluorescence could significantly help community clinicians in making wise decisions^17^. Subsequently, a similar prospective study was conducted by Bhatia *et al*. in general practice, in which a more detailed decision-making protocol was designed, including background information, COE, autofluorescence examination, combined examination, review appointment and referral appointment. Consequently, the combination of autofluorescence with COE achieved a dramatic increase in sensitivity and specificity compared to autofluorescence alone, as 73.9% and 97.9% rather than 64% and 54.3%, respectively. However, as it was only a single-center study, multi-center research on this protocol is needed to further verify its potential^35^.

Moreover, diascopic fluorescence has been shown to decrease the FP results caused by inflammation in previous studies^29^. Farah *et al*. found that complete blanching appeared in 10 dysplastic lesions and 1 OSCC, but the pressure and tool were both to blame^29^. In Bhatia’s study, they used diascopic fluorescence in general practice firstly and suggested that the back of a periodontal or sickle probe may be more proper for the test and that these lesions of partial blanching had the highest rate of referrals^35^.

The limitations of this study are as follows. First, failure to include letters to the editors and ongoing studies may cause publication bias. Second, as our included studies applying autofluorescence for the diagnosis of OSCC and OPMD were based on patients of superior clinics rather than primary care, overestimation of its diagnostic accuracy may be a concern. More well-designed clinical trials about its application in general practice are strongly encouraged to support its use by general dentists. Third, owing to the lack of necessary data in our included studies, we are unable to perform a subgroup analysis about whether factors such as age, race were the possible cause of heterogeneity. Lastly, with the advent of various non-invasive diagnostic tools, including chemiluminescence, oral CDx brush biopsy, toluidine blue and narrow band imaging (NBI) being used in oral clinics for OSCC and OPMD^10^, it may be necessary for us to compare the accuracy of autofluorescence with those approaches in the future, significant to help select more efficacious early diagnostic tool for OSCC and OPMD.

In conclusion, autofluorescence is a promising non-invasive tool with relatively high accuracy for the early diagnosis of OSCC and OPMD. It also presents with good application stability for detecting lesions of 5 anatomical parts. In light of its medium specificity when used alone, it is more reliable to serve as an adjunct in the hands of oral specialists. However, in an attempt to facilitate its use in primary care to improve the survival rates of OSCC, some promising approaches could be adopted, including its use in combination with proper algorithms.

## Additional Information

**How to cite this article**: Luo, X. *et al*. Accuracy of autofluorescence in diagnosing oral squamous cell carcinoma and oral potentially malignant disorders: a comparative study with aero-digestive lesions. *Sci. Rep.* **6**, 29943; doi: 10.1038/srep29943 (2016).

## Supplementary Material

Supplementary Information

## Figures and Tables

**Table 1:** Summary of the main characteristics of studies that applying autofluorescence to detect OSCC and OPMD.

First Author	Year	Country	Sample Size	TP	FP	FN	TN
Onizawa K^48^	1996	Japan	32	14	1	2	15
Gillenwater A^49^	1998	USA	28	16	0	1	11
Wang CY^50^	1999	China	32	13	1	3	15
Onizawa K^51^	1999	Japan	124	75	2	5	42
van Staveren HJ^52^	2000	Netherlands	28	19	0	3	6
Wang CY^53^	2003	China	97	21	3	5	68
Muller MG^54^	2003	USA	74	27	1	2	44
Majumder SK^55^	2003	India	325	78	9	5	233
Svistun E^56^	2004	USA	23	15	1	1	6
Majumder SK^57^	2005	India	325	74	16	9	146
Poh CF^16^	2006	Canada	122	52	4	1	65
Lane PM^21^	2006	Canada	50	43	0	1	6
Jayaprakash V^58^	2009	USA	249	123	40	47	39
Roblyer D^20^	2009	USA	159	69	5	2	83
Koch FP^59^	2010	Germany	78	31	37	2	8
Mehrotra R^60^	2010	India	156	6	88	6	56
Awan K.H.^19^	2011	United Kingdom	116	37	61	7	11
Scheer M^61^	2011	Germany	64	12	10	0	42
Matsumoto K^62^	2011	Japan	74	39	8	25	2
Farah CS^29^	2012	Australia	118	8	34	19	57
Hanken H^63^	2013	Germany	60	47	8	1	4
Jayaprakash V^64^	2013	USA	255	164	59	20	12
Piazza C^65^	2013	Italy	116	66	13	18	19
Petruzzi M^66^	2014	Italy	56	21	11	9	15

TP: true positive; FP: false positive; FN: false negative; TN: true negative; OSCC: oral squamous cell carcinoma; OPMD: oral potentially malignant disorders.

**Table 2:** Summary of the methodological quality of the included studies that employing autofluorescence to identify OSCC and OPMD according to QUADAS-2 criteria.

Studies	Risk of Bias				Applicability Concerns		
	Patient Selection	Index Test	Reference Standard	Flow and Timing	Patient Selection	Index Test	Reference Standard
Onizawa K-1996	UR	LR	LR	LR	LC	LC	LC
Gillenwater A-1998	LR	LR	LR	LR	LC	LC	LC
Wang CY-1999	HR	UR	LR	LR	UC	UC	LC
Onizawa K-1999	UR	UR	HR	UR	LC	LC	LC
Van Staveren HJ-2000	LR	LR	LR	LR	LC	LC	LC
Wang CY-2003	LR	LR	LR	LR	LC	LC	LC
Muller MG-2003	HR	LR	LR	HR	UC	UC	LC
Majumder SK-2003	LR	LR	LR	LR	LC	LC	LC
Svistun E-2004	UR	LR	LR	LR	LC	LC	LC
Majumder SK-2005	LR	LR	LR	LR	LC	LC	LC
Poh CF-2006	HR	LR	LR	LR	UC	LC	LC
Lane PM-2006	HR	LR	LR	LR	UC	LC	LC
Jayaprakash V-2009	HR	LR	LR	LR	UC	LC	LC
Roblyer D-2009	LR	LR	LR	LR	LC	LC	LC
Koch FP-2010	UR	UR	LR	LR	LC	LC	LC
Mehrotra R-2010	HR	LR	LR	UR	UC	LC	LC
Awan K.H. -2011	LR	UR	UR	HR	LC	LC	LC
Scheer M-2011	HR	UR	LR	LR	UC	LC	LC
Matsumoto K-2011	LR	LR	LR	LR	LC	LC	LC
Farah CS-2012	HR	LR	LR	UR	UC	LC	LC
Hanken H-2013	LR	LR	LR	LR	LC	LC	LC
Jayaprakash V-2013	UR	UR	LR	LR	LC	LC	LC
Piazza C-2013	UR	UR	LR	LR	LC	LC	LC
Petruzzi M-2014	LR	LR	LR	UR	LC	LC	LC

LR: low risk; HR: high risk; UR: unclear risk; LC: low concern; HC: high concern; UC: unclear concern; OSCC: oral squamous cell carcinoma; OPMD: oral potentially malignant disorders; QUADAS-2: quality assessment for studies of diagnostic accuracy.

**Table 3:** Pooled estimates of diagnostic indexes regarding the accuracy of autofluorescence for the PML and ML of the oral cavity, lung, esophagus, stomach, and colorectum.

Test	No. of studies	No. of samples	sensitivity	Pooled estimates (95% CI)				
				specificity	PLR	NLR	DOR	AUC
Oral cavity	24	2761	0.89 (0.82–0.93)	0.80 (0.64–0.90)	4.54 (2.28–9.04)	0.14 (0.08–0.24)	32.37 (10.47–100.12)	0.92 (0.89–0.94)
Lung	25	4384	0.89 (0.86–0.92)	0.63 (0.52–0.73)	2.43 (1.82–3.24)	0.17 (0.13–0.22)	14.52 (9.25–22.81)	0.89 (0.86–0.91)
Esophagus	12	2514	0.78 (0.57–0.91)	0.77 (0.58–0.89)	3.43 (1.81–6.50)	0.28 (0.14–0.58)	12.17 (4.32–34.27)	0.85 (0.81–0.87)
Stomach	9	2115	0.88 (0.76–0.94)	0.73 (0.50–0.88)	3.28 (1.52–7.08)	0.17 (0.07–0.39)	19.31 (4.49–83.05)	0.89 (0.86–0.92)
Colorectum	19	2904	0.91 (0.86–0.94)	0.78 (0.64–0.88)	4.15 (2.40–7.17)	0.11 (0.07–0.19)	36.27 (14.20–92.63)	0.93 (0.91–0.95)

PML: premalignant lesions; ML: malignant lesions; CI: confidence interval; PLR: positive likelihood ratio; NLR: negative likelihood ratio; DOR: diagnostic odd ratio; AUC: area under the curve.

**Table 4:** Pooled estimates of diagnostic indexes regarding the accuracy of using autofluorescence alone or with algorithms for detecting OSCC and OPMD.

Test	No. of studies	No. of samples	sensitivity	Pooled estimates (95%CI)				
				specificity	PLR	NLR	DOR	AUC
(A) AF only	16	1693	0.88 (0.77–0.94)	0.62 (0.41–0.79)	2.31 (1.31–4.09)	0.20 (0.09–0.46)	11.52 (3.10–42.87)	0.85 (0.82–0.88)
(B) AF with algorithms	8	1068	0.92 (0.88–0.94)	0.95 (0.92–0.97)	17.28 (10.96–27.22)	0.09 (0.06–0.13)	194.11 (94.27–399.71)	0.98 (0.96–0.99)
*u* test of A and B (*p* value)	—	—	NS	*	*	NS	*	*

OSCC: oral squamous cell carcinoma; OPMD: oral potentially malignant disorders; CI: confidence interval; PLR: positive likelihood ratio; NLR: negative likelihood ratio; DOR: diagnostic odd ratio; AUC: area under the curve; AF: autofluorescence;

NS: non-significant (*p* > 0.05); *p < 0.05.
