# Supplementary material for: Accuracy of autofluorescence in diagnosing oral squamous cell carcinoma and oral potentially malignant disorders: a comparative study with aero-digestive lesions
Source: Sci Rep. 2016 Jul 15;6:29943. doi: 10.1038/srep29943 (PMC4945954; doi:10.1038/srep29943)
Supplement: Supplementary Information [file srep29943-s1.pdf]

Accuracy of autofluorescence in diagnosing oral squamous cell carcinoma and oral potentially malignant disorders: a comparative study with aero-digestive lesions

Xiaobo Luo[1,§], Hao Xu[1,§], Mingjing He[1], Qi Han[1], Hui Wang[2], Chongkui Sun[1], Jing Li[1], Lu Jiang[1], Yu Zhou[1], Hongxia Dan[1], Xiaodong Feng[1], Xin Zeng[1,*] & Qianming Chen[1,*]

[1] State Key Laboratory of Oral Diseases, West China Hospital of Stomatology, Sichuan University, Chengdu, Sichuan 610041, China

[2] Department of Oral Medicine, School of Stomatology, Capital Medical University, Beijing 100050, China

[§] These authors contributed equally to this work.

[*] Corresponding authors:
1. Xin Zeng, Ph.D & DDS. State Key Laboratory of Oral Diseases, West China Hospital of Stomatology, Sichuan University, No. 14, Sec.3, RenMinNan Road, Chengdu, Sichuan, 610041, China.
Fax: +86-28-855503484; E-mail: zengxin22@163.com
2. Qianming Chen, Ph.D & DDS. State Key Laboratory of Oral Diseases, West China Hospital of Stomatology, Sichuan University, No. 14, Sec.3, RenMinNan Road, Chengdu, Sichuan, 610041, China. Fax: +86-28-85405251; E-mail: qmchen@scu.edu.cn

Supplementary Figures

Supplementary Figure S1: Flow diagram showing the selection processes of eligible articles that using autofluorescence to diagnose PML and ML of the lung (a), esophagus (b), stomach (c), and colorectum (d).

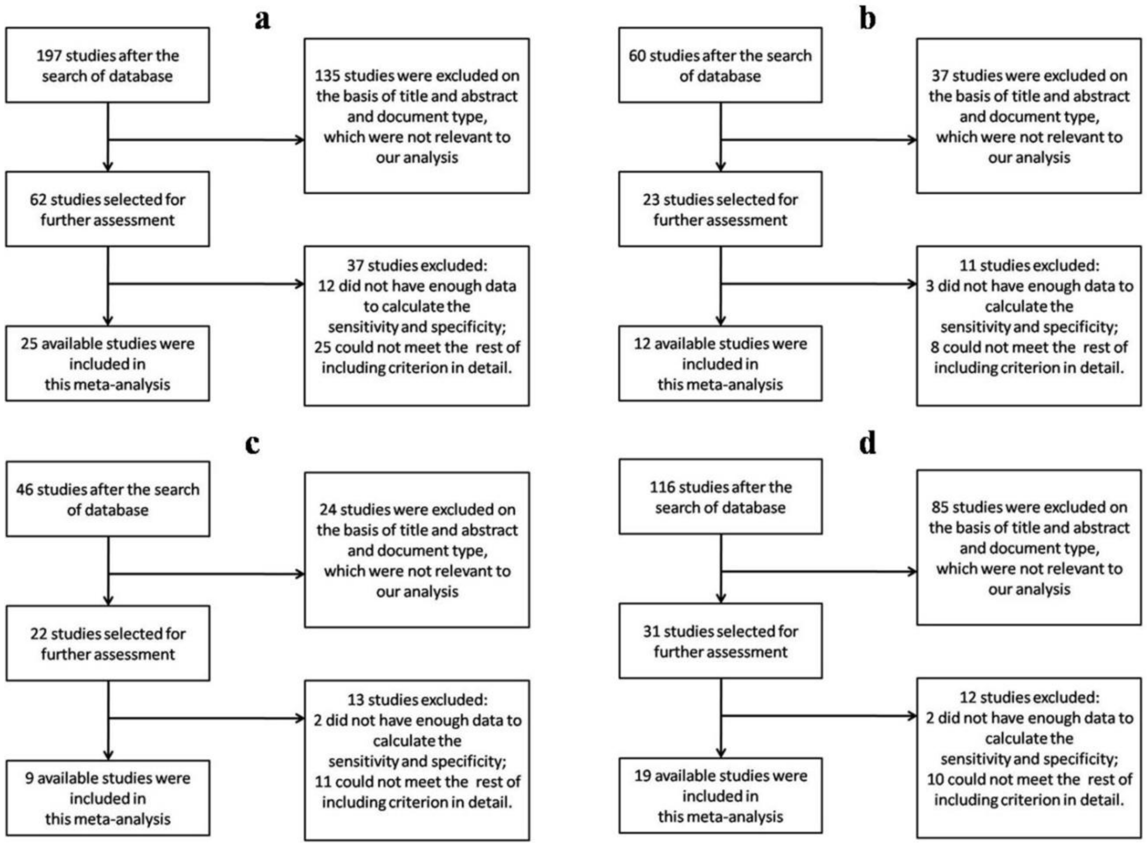

PML: premalignant lesions; ML: malignant lesions.

Supplementary Figure S2: Forest plots of the sensitivity and specificity of studies that employing autofluorescence in the diagnosis of PML and ML of the lung (a), esophagus (b), stomach (c), and colorectum (d).

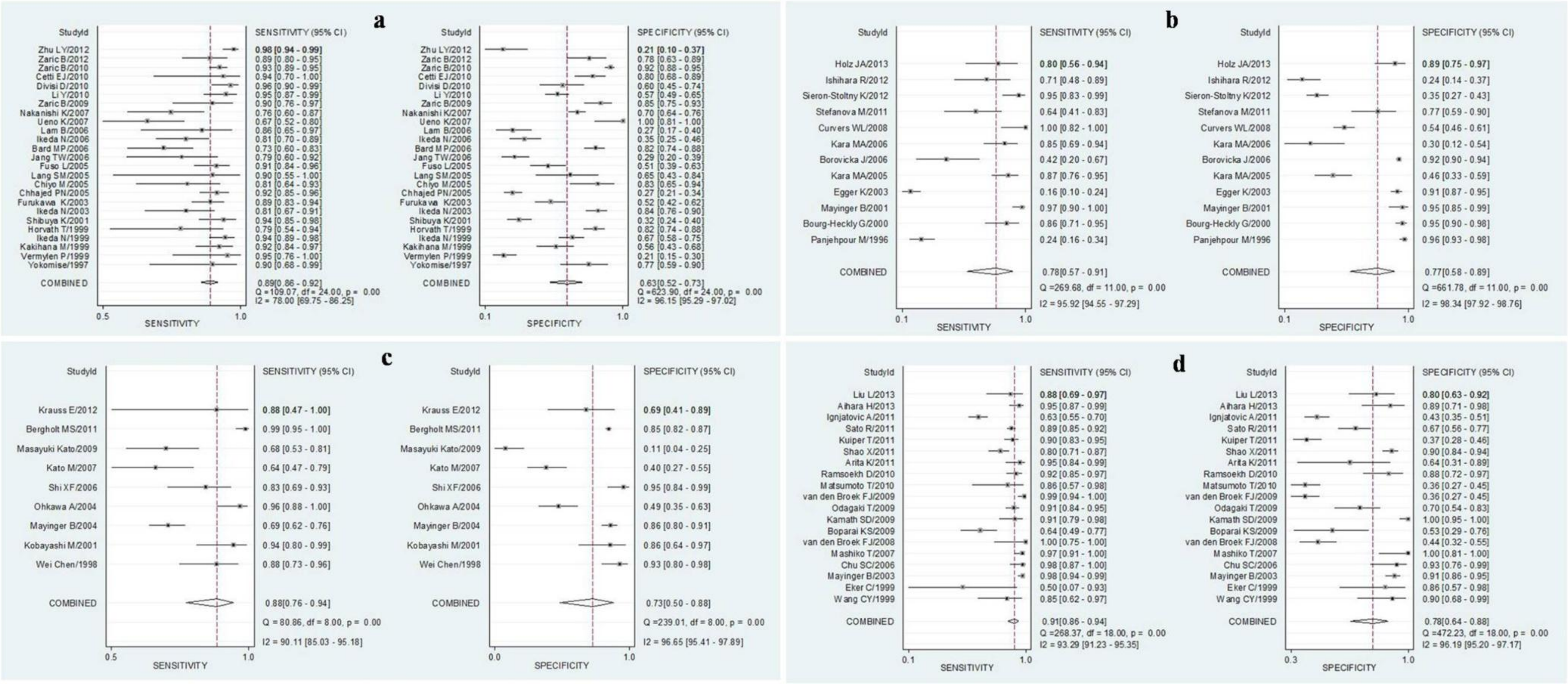

PML: premalignant lesions; ML: malignant lesions.

Supplementary Figure S3: Forest plots of the sensitivity and specificity of studies using autofluorescence alone for the detection of OSCC and OPMD.

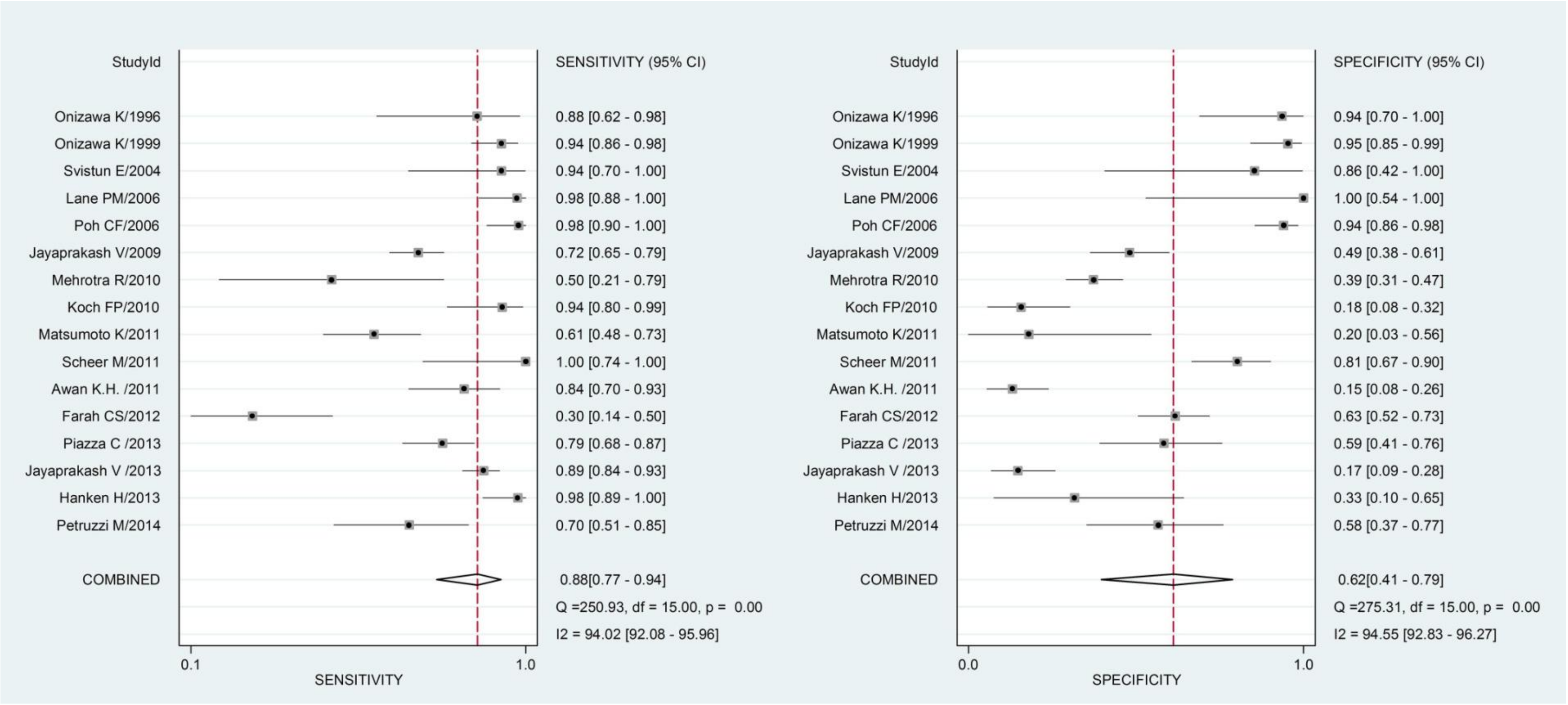

OSCC: oral squamous cell carcinoma; OPMD: oral potentially malignant disorders.

Supplementary Figure S4: Forest plots of the sensitivity and specificity of studies using autofluorescence combined with algorithms for detecting OSCC and OPMD.

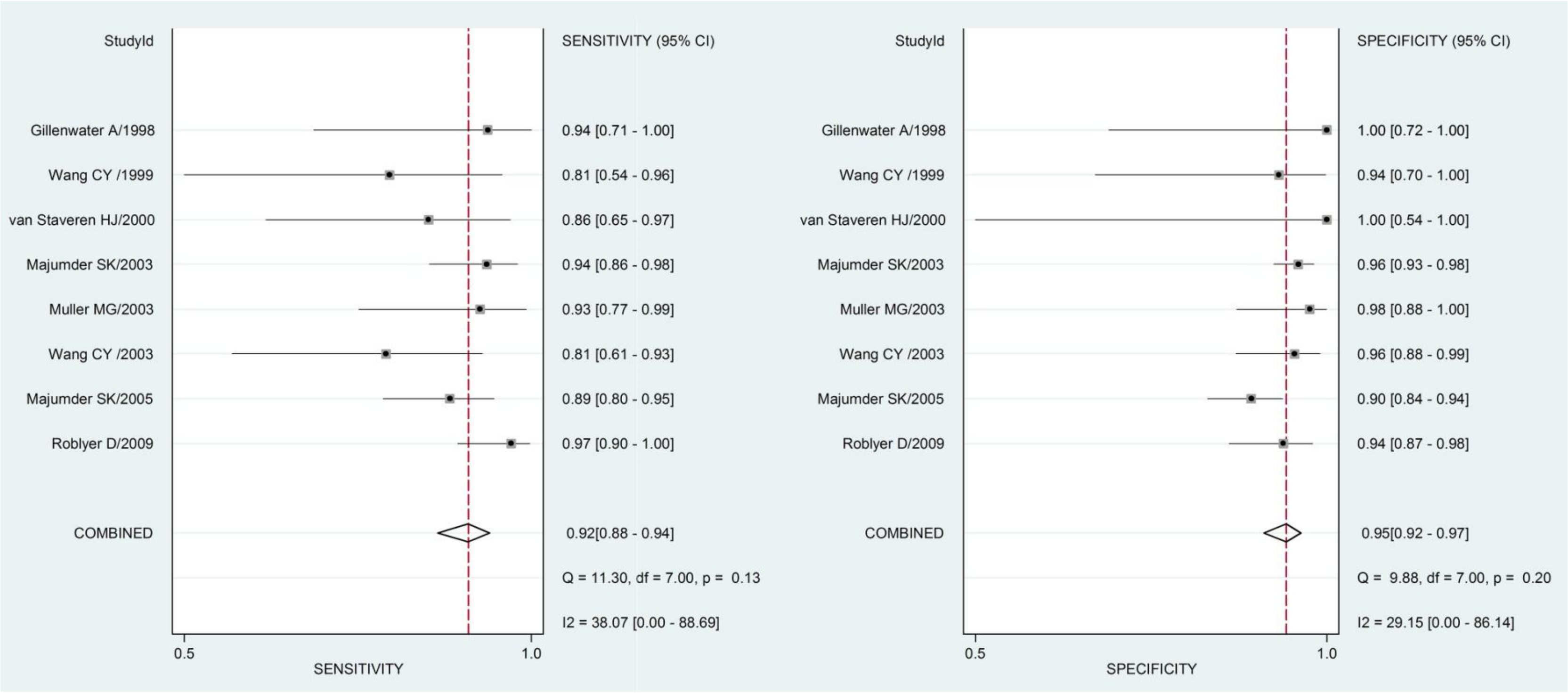

OSCC: oral squamous cell carcinoma; OPMD: oral potentially malignant disorders.

Supplementary Figure S5: HSROC curve of studies that utilizing autofluorescence for identifying PML and ML of the lung (a), esophagus (b), stomach (c), and colorectum (d).

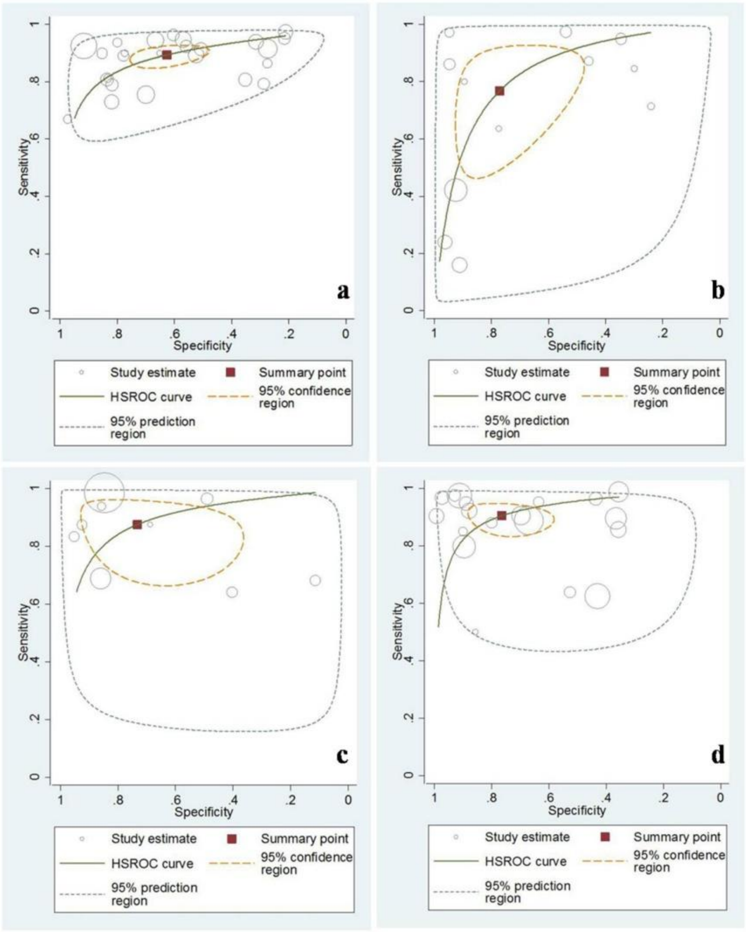

HSROC: Hierarchical summary receiver operating characteristic; PML: premalignant lesions; ML: malignant lesions.

Supplementary Figure S6: The wind rose picture revealing the overall diagnostic accuracy of autofluorescence for PML and ML of the oral cavity, lung, esophagus, stomach, and colorectum.

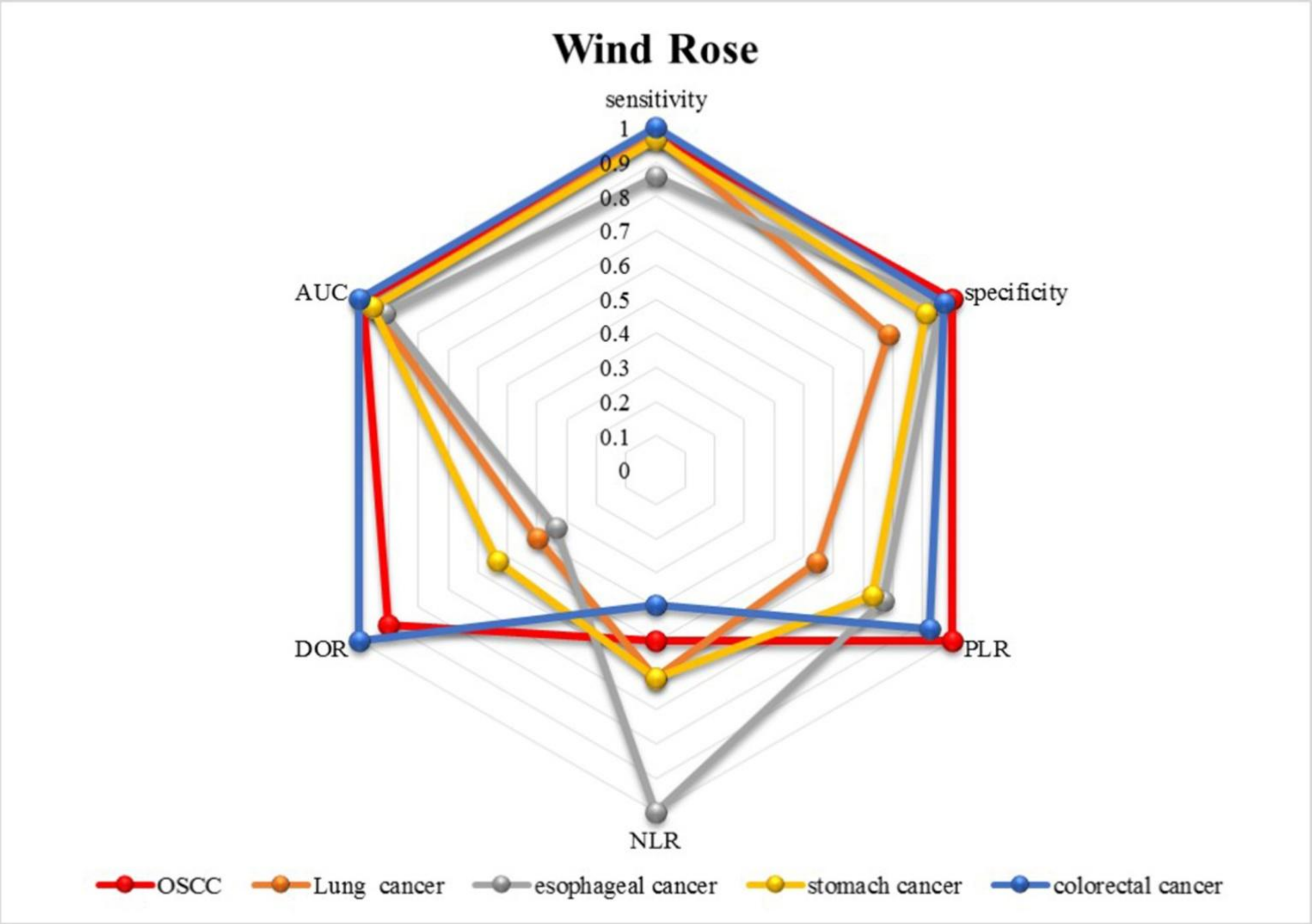

PML: premalignant lesions; ML: malignant lesions.

Supplementary Figure S7: Begg's funnel plot for the evaluation of potential publication bias of studies that applying autofluorescence to detect PML and ML of the lung (a), esophagus (b), stomach (c), and colorectum (d).

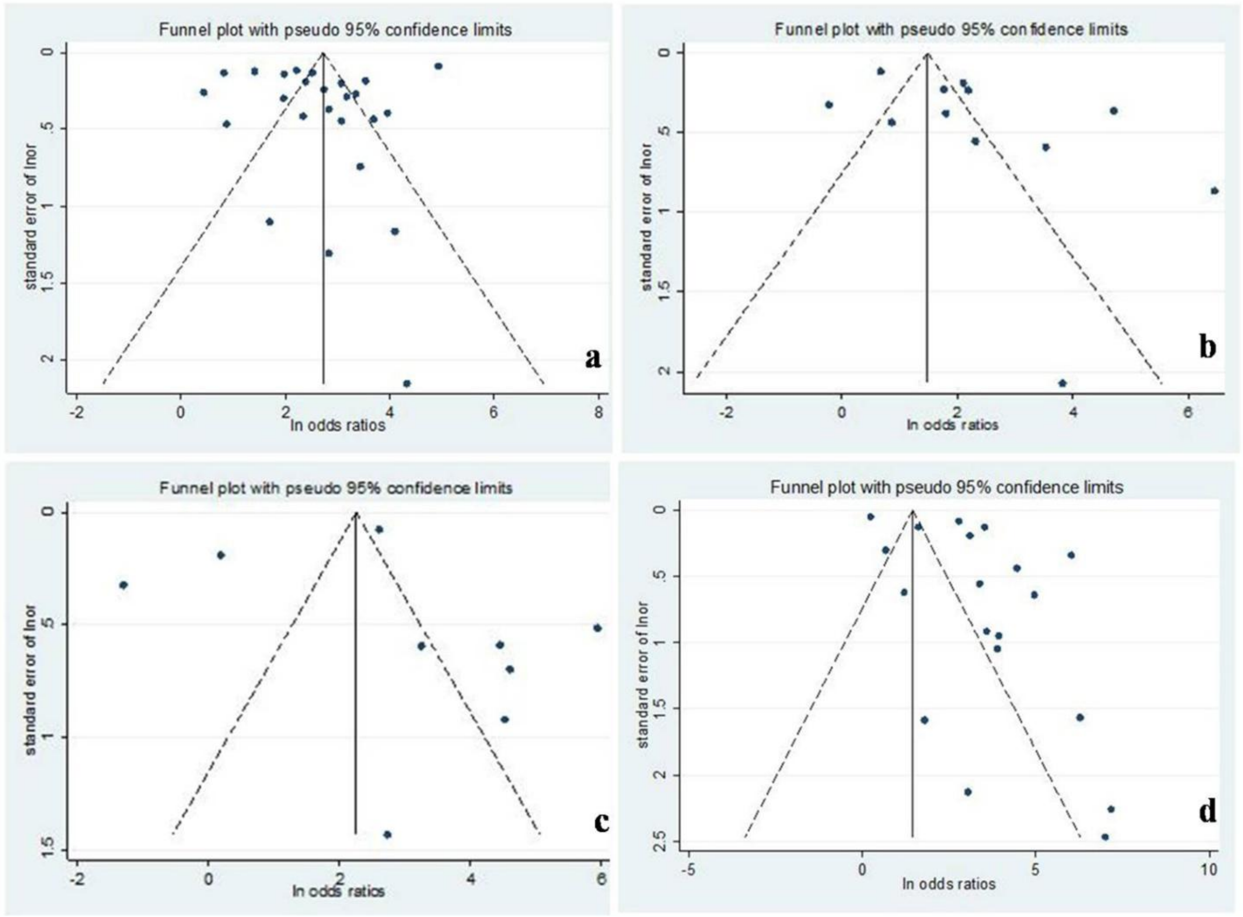

PML: premalignant lesions; ML: malignant lesions.

Supplementary Tables

Supplementary Table S1. Summary of the main characteristics of studies that applying autofluorescence to detect PML and ML of the lung, esophagus, stomach, and colorectum

First Author	Year	Country	Detecting Site	Sample Size	TP	FP	FN	TN
Yokomise H[67]	1997	Japan	lung	51	18	7	2	24
Vermylen P[68]	1999	Belgium	lung	142	20	95	1	26
Kakihana M[69]	1999	Japan	lung	147	73	30	6	38
Ikeda N[70]	1999	Japan	lung	262	120	45	7	90
Horvath T[71]	1999	Czech	lung	146	15	23	4	104
Shibuya K[72]	2001	Japan	lung	212	62	100	4	46
Ikeda N[73]	2003	Japan	lung	163	38	19	9	97
Furukawa K[74]	2003	Japan	lung	234	117	49	14	54
Chhajed PN[75]	2005	Japan	lung	343	120	154	11	58

Chiyo M[76]	2005	Japan	lung	62	26	5	6	25
Lang SM[77]	2005	Germany	lung	33	9	8	1	15
Fuso L[78]	2005	Italy	lung	166	85	36	8	37
Jang TW[79]	2006	Korea	lung	130	23	72	6	29
Bard MP[80]	2006	Netherlands	lung	191	46	23	17	105
Ikeda N[81]	2006	Japan	lung	166	63	57	15	31
Lam B[82]	2006	China	lung	84	19	45	3	17
Ueno K[83]	2007	Japan	lung	64	31	0	15	18
Nakanishi K[84]	2007	Japan	lung	288	34	73	11	170
Zaric B[85]	2009	Serbia	lung	108	36	10	4	58
Li Y[86]	2010	China	lung	241	72	71	4	94
Divisi D[22]	2010	England	lung	130	79	19	3	29
Cetti EJ[87]	2010	Switzerland	lung	81	15	13	1	52

Zaric B[88]	2010	Serbia	lung	624	286	26	23	289
Zaric B[89]	2012	Serbia	lung	118	65	10	8	35
Zhu LY[90]	2012	China	lung	198	156	30	4	8
Panjehpour M[91]	1996	US	esophagus	308	22	8	70	208
Bourg-Heckly G[92]	2000	France	esophagus	187	31	8	5	143
Mayinger B[23]	2001	Germany	esophagus	129	70	3	2	54
Egger K[93]	2003	Germany	esophagus	345	19	20	100	206
Kara MA[94]	2005	Netherlands	esophagus	116	48	33	7	28
Borovicka J[95]	2006	Switzerland	esophagus	785	8	58	11	708
Kara MA[96]	2006	Netherlands	esophagus	59	33	14	6	6
Curvers WL[97]	2008	Netherlands	esophagus	199	19	83	0	97
Stefanova M[98]	2011	Czech Republic	esophagus	53	14	7	8	24
Sieron-Stoltny K[99]	2012	Poland	esophagus	196	38	102	2	54

Ishihara R[100]	2012	Japan	esophagus	79	15	44	6	14
Holz JA[101]	2013	Netherlands	esophagus	58	16	4	4	34
Chen W[102]	1998	China	stomach	80	35	3	5	37
Kobayashi M[103]	2001	Japan	stomach	54	31	3	2	18
Mayinger B[104]	2004	Germany	stomach	344	124	23	56	141
Ohkawa A[105]	2004	Japan	stomach	109	54	27	2	26
Shi XF[106]	2006	China	stomach	84	35	2	7	40
Kato M[24]	2007	Japan	stomach	91	25	31	14	21
Kato M[107]	2009	Japan	stomach	91	32	39	15	5
Bergholt MS[108]	2011	Singapore	stomach	1238	138	168	2	930
Krauss E[109]	2012	Germany	stomach	24	7	5	1	11
Wang CY[110]	1999	China	colorectum	40	17	2	3	18
Eker C[111]	1999	Sweden	colorectum	18	2	2	2	12

Mayinger B[25]	2003	Germany	colorectum	311	157	13	4	137
Chu SC[112]	2006	China	colorectum	70	41	2	1	26
Mashiko T[113]	2007	Japan	colorectum	94	74	0	2	18
van den Broek FJ[114]	2008	Netherlands	colorectum	91	13	44	0	34
Boparai KS[115]	2009	Netherlands	colorectum	66	30	9	17	10
Kamath SD[116]	2009	India	colorectum	115	42	0	4	69
van den Broek FJ[117]	2009	Netherlands	colorectum	208	89	76	1	42
Matsumoto T[118]	2010	Japan	colorectum	126	12	72	2	40
Ramsoekh D[119]	2010	Netherlands	colorectum	123	83	4	7	29
Arita K[120]	2011	Japan	colorectum	54	41	4	2	7
Shao X[121]	2011	Singapore	colorectum	263	79	17	20	147
Kuiper T[122]	2011	Netherlands	colorectum	239	104	78	12	45
Sato R[123]	2011	Japan	colorectum	424	301	28	38	57

Ignjatovic A[124]	2011	UK	colorectum	320	100	91	60	69
Odagaki T[125]	2013	Japan	colorectum	180	124	13	13	30
Aihara H[126]	2013	Japan	colorectum	102	71	3	4	24
Liu L[127]	2013	China	colorectum	60	22	7	3	28

PML: premalignant lesions; ML: malignant lesions; TP: true positive; FP: false positive; FN: false negative; TN: true negative.

Supplementary Table S2. Summary of the methodological quality of the included studies that employing autofluorescence to identifiy PML and ML of the lung, esophagus, stomach, and colorectum according to QUADAS-2 criteria

Studies	Detecting Site	Risk of Bias				Applicability Concerns		
		Patient Selection	Index Test	Reference Standard	Flow and Timing	Patient Selection	Index Test	Reference Standard
Yokomise H-1997	lung	LR	LR	LR	LR	UC	LC	LC
Vermylen P-1999	lung	LR	LR	LR	LR	UC	LC	LC
Kakihana M-1999	lung	LR	LR	LR	LR	LC	LC	LC
Ikeda N-1999	lung	LR	LR	LR	LR	LC	LC	LC
Horvath T-1999	lung	LR	LR	LR	LR	LC	LC	LC
Shibuya K-2001	lung	LR	LR	LR	LR	LC	LC	LC
Ikeda N-2003	lung	LR	LR	LR	LR	UC	LC	LC
Furukawa K-2003	lung	LR	UR	UR	LR	LC	LC	LC

Chhajed PN-2005	lung	LR	LR	LR	LR	LC	LC	LC
Chiyo M-2005	lung	LR	LR	LR	LR	UC	LC	LC
Lang SM-2005	lung	LR	LR	LR	LR	UC	LC	LC
Fuso L-2005	lung	LR	UR	UR	LR	LC	LC	LC
Jang TW-2006	lung	LR	LR	LR	LR	LC	LC	LC
Bard MP-2006	lung	LR	LR	LR	LR	LC	LC	LC
Ikeda N-2006	lung	LR	LR	LR	LR	LC	LC	LC
Lam B-2006	lung	LR	LR	LR	LR	LC	LC	LC
Ueno K-2007	lung	LR	UR	UR	LR	LC	LC	LC
Nakanishi K-2007	lung	LR	LR	LR	LR	UC	LC	LC
Zaric B-2009	lung	LR	LR	LR	LR	UC	LC	LC
Li Y-2010	lung	LR	UR	UR	LR	UC	LC	LC
Divisi D-2010	lung	LR	UR	UR	LR	LC	LC	LC

Cetti EJ-2010	lung	LR	LR	LR	LR	UC	LC	LC
Zaric B-2010	lung	LR	LR	LR	LR	UC	LC	LC
Zaric B-2012	lung	LR	LR	LR	LR	UC	LC	LC
Zhu LY-2012	lung	LR	UR	UR	LR	UC	LC	LC
Panjehpour M-1996	esophagus	HR	LR	LR	LR	LC	LC	LC
Bourg-Heckly G-2000	esophagus	HR	UR	UR	LR	LC	LC	LC
Mayinger B-2001	esophagus	LR	LR	LR	LR	UC	LC	LC
Egger K-2003	esophagus	LR	LR	LR	LR	LC	LC	LC
Kara MA-2005	esophagus	LR	LR	LR	LR	LC	LC	LC
Borovicka J-2006	esophagus	LR	LR	LR	LR	LC	LC	LC
Kara MA-2006	esophagus	LR	LR	LR	LR	UC	LC	LC
Curvers WL-2008	esophagus	LR	LR	LR	LR	LC	LC	LC

Stefanova M-2011	esophagus	LR	LR	LR	LR	LC	LC	LC
Sieron-Stoltny K-2012	esophagus	HR	UR	UR	LR	LC	LC	LC
Ishihara R-2012	esophagus	LR	LR	LR	LR	UC	LC	LC
Holz JA-2013	esophagus	LR	UR	UR	HR	LC	LC	LC
Chen W-1998	stomach	LR	UR	UR	HR	UC	LC	LC
Kobayashi M-2001	stomach	HR	UR	UR	LR	UC	LC	LC
Mayinger B-2004	stomach	LR	LR	LR	LR	UC	LC	LC
Ohkawa A-2004	stomach	LR	LR	LR	LR	LC	LC	LC
Shi XF-2006	stomach	LR	UR	UR	LR	UC	LC	LC
Kato M-2007	stomach	LR	LR	LR	LR	LC	LC	LC
Kato M-2009	stomach	LR	LR	LR	LR	LC	LC	LC
Bergholt MS-2011	stomach	LR	LR	LR	LR	LC	LC	LC

Krauss E-2012	stomach	LR	LR	LR	LR	LC	LC	LC
Wang CY-1999	colorectum	LR	LR	LR	LR	UC	LC	LC
Eker C-1999	colorectum	LR	LR	LR	LR	LC	LC	LC
Mayinger B-2003	colorectum	LR	LR	LR	LR	LC	LC	LC
Chu SC-2006	colorectum	HR	LR	LR	LR	LC	LC	LC
Mashiko T-2007	colorectum	HR	LR	LR	LR	LC	LC	LC
van den Broek FJ-2008	colorectum	LR	LR	LR	LR	LC	LC	LC
Boparai KS-2009	colorectum	LR	LR	LR	LR	LC	LC	LC
Kamath SD-2009	colorectum	LR	UR	UR	LR	LC	LC	LC
van den Broek FJ-2009	colorectum	LR	LR	LR	LR	LC	LC	LC

Matsumoto T-2010	colorectum	LR	LR	LR	LR	LC	LC	LC
Ramsoekh D-2010	colorectum	LR	LR	LR	LR	UC	LC	LC
Arita K-2011	colorectum	LR	LR	LR	LR	LC	LC	LC
Shao X-2011	colorectum	LR	UR	UR	LR	LC	LC	LC
Kuiper T-2011	colorectum	LR	LR	LR	LR	LC	LC	LC
Sato R-2011	colorectum	LR	LR	LR	LR	LC	LC	LC
Ignjatovic A-2011	colorectum	LR	LR	LR	LR	LC	LC	LC
Odagaki T-2013	colorectum	LR	UR	UR	LR	LC	LC	LC
Aihara H-2013	colorectum	LR	UR	UR	LR	LC	LC	LC
Liu L-2013	colorectum	LR	UR	UR	LR	UC	LC	LC

LR: low risk; HR: high risk; UR: unclear risk; LC: low concern; HC: high concern; UC: unclear concern; PML: premalignant lesions; ML: malignant lesions; QUADAS-2: quality assessment for studies of diagnostic accuracy.
